# Biomarkers in Pancreatic Cancer as Analytic Targets for Nanomediated Imaging and Therapy

**DOI:** 10.3390/ma14113083

**Published:** 2021-06-04

**Authors:** Cristiana Maria Grapa, Lucian Mocan, Dana Crisan, Mira Florea, Teodora Mocan

**Affiliations:** 1 Physiology Department, “Iuliu Hatieganu” University of Medicine and Pharmacy, 400126 Cluj-Napoca, Romania; grapa.cristiana.maria@umfcluj.ro (C.M.G.); teodora.mocan@umfcluj.ro (T.M.); 2 Nanomedicine Department, Regional Institute of Gastroenterology and Hepatology, 400126 Cluj-Napoca, Romania; 3 3-rd Surgery Clinic, University of Medicine and Pharmacy, 400126 Cluj-Napoca, Romania; 4 Internal Medicine Department, “Iuliu Hatieganu” University of Medicine and Pharmacy, 400126 Cluj-Napoca, Romania; crisan.dc@gmail.com; 5 Family Medicine Department, Iuliu Hatieganu University of Medicine and Pharmacy, 400126 Cluj-Napoca, Romania; miraflorea@umfcluj.ro

**Keywords:** biomarkers, nanoparticles, pancreatic cancer, targeted therapy

## Abstract

As the increase in therapeutic and imaging technologies is swiftly improving survival chances for cancer patients, pancreatic cancer (PC) still has a grim prognosis and a rising incidence. Practically everything distinguishing for this type of malignancy makes it challenging to treat: no approved method for early detection, extended asymptomatic state, limited treatment options, poor chemotherapy response and dense tumor stroma that impedes drug delivery. We provide a narrative review of our main findings in the field of nanoparticle directed treatment for PC, with a focus on biomarker targeted delivery. By reducing drug toxicity, increasing their tumor accumulation, ability to modulate tumor microenvironment and even improve imaging contrast, it seems that nanotechnology may one day give hope for better outcome in pancreatic cancer. Further conjugating nanoparticles with biomarkers that are overexpressed amplifies the benefits mentioned, with potential increase in survival and treatment response.

## 1. Introduction

All studies involving pancreatic cancer start with the same harrowing observation: this type of cancer has one of the worst outcomes, with high morbidity and mortality, its survival rate being lower than 10% [1]. It is expected that by the year 2030, PC will be the second cause of cancer-related deaths [2]. Regarding its histology, 90% of all tumors are pancreatic adenocarcinomas (PDAC), which arise from the ductal epithelium of the exocrine pancreas. Its long asymptomatic state and its rapid growth, along with poor treatment response are responsible for a median survival of 5 to 8 months, following diagnosis [3].

The only curative treatment is represented by surgery, but only about 20% benefit from it, as PC is often asymptomatic and most of them are diagnosed in late stages; furthermore, even for patients who undergo surgical treatment, up to 80% still progress to local recurrence or metastases [4]. Chemotherapy regimens available are represented by gemcitabine as first line treatment; FOLFIRINOX (a combination of four chemotherapeutics: follinic acid,5-fluorouracil, oxaliplatin and irinotecan) and recently, albumin bound paclitaxel [5]. Liposomal irinotecan was also approved as of late for patients with advanced disease, but due to its important toxicity, its use in current clinical practice is being questioned [6].

Intrinsic barriers such as drug resistance and extrinsic cell barriers, mainly represented by the tumor microenvironment, need to be overcome in order to properly manage this type of cancer. Current treatment modalities are insufficient, thus, effort was put into developing new and improved therapeutic strategies. Nanotechnology plays an important part in the development of tumor targeted therapies; nanoparticles, through their small size, ability to breach tumor barriers and gather into the neoplastic tissue hold great promise for overcoming obstacles in pancreatic cancer treatment [7]. Moving forward, nanoparticle targeted therapy can further increase their prospective use.

Aside from carbohydrate antigen CA 19-9, a biomarker which is not completely specific to pancreatic cancer, no other biomarker has been approved for diagnosis, prognosis or early detection in PC. Early screening for PC using imaging techniques, like computer tomography (CT) or magnetic resonance (MRI) are not recommended, mainly due to cost-efficiency and their inability to detect pancreatic lesions smaller than 5–8 mm [8].

Recent studies have demonstrated the potential use of a multitude of biomarkers for early detection, prognosis or treatment follow-up, although larger validation studies are required. Nanoparticle targeted therapies using biomarkers is a rapidly evolving field of research. Multiple types of nanoparticles such as magnetic iron oxide nanoparticles, single wall carbon nanotubes and others [9,10] have been used on different pancreatic cancer cell lines along with biomarkers which proved to be beneficial in augmenting the nanoconjugates therapeutic efficacy. The biomarkers used appeared to enhance either accumulation of the nanoparticles used, the chemotherapy effectiveness or contrast imaging, proving that despite the fact that there is still a long way to go until clinical implementation, steps are made in the right direction. The main purpose of our research is to emphasize the potential impact of nanotechnology in pancreatic cancer, a type of cancer with very limited therapy options.

In this narrative review, we provide an outline of the main therapeutic obstacles in pancreatic cancer, and the potential use of nanotechnology and biomarker targeted therapy for diminishing the burden of this disease. We searched the Medline/PubMed database for eligible articles using specific keywords like “pancreatic cancer”, “stroma”, “biomarker”, “nanoparticle”, “nanotechnology” and “targeted therapy” together or in combinations. We selected the articles based on their relevance for our desired approach and included the ones that met our criteria: nanotechnology and its involvement in pancreatic cancer therapy. Only English written articles were included; almost all articles were published from January 2001 to February 2021, with few exceptions we could not exclude because of their significance.

## 2. Results and Discussions

### 2.1. Pancreatic Tumor Microenvironment and Therapeutic Challenges

**Figure 1:**
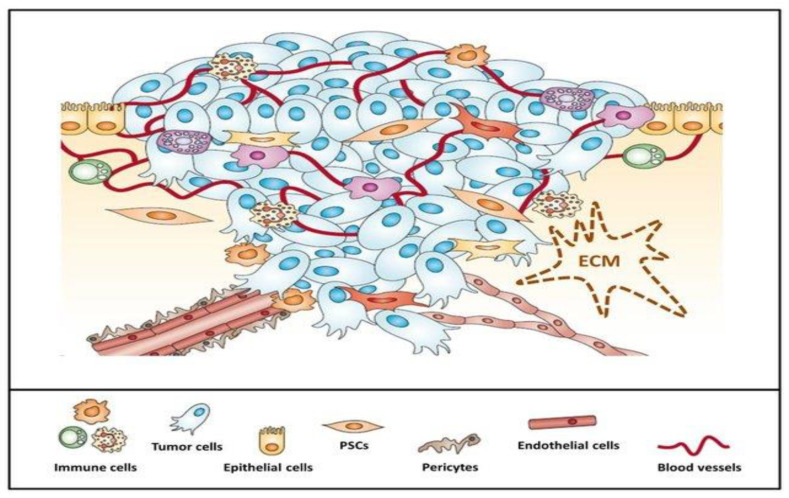
Pancreatic tumor microenvironment is composed of neoplastic cells surrounded by the abundant stroma at the cells that contribute to its development: pancreatic stellate cells (PSCs), immune cells such as neutrophils or lymphocytes, epithelial and endothelial cells and pericytes. Reprinted with permission from [24].

An abundant number of new diagnostic and therapeutic prospects emerged in the past years involving different types of cancer; regrettably, PDAC prognosis remains grim. The main reason is thought to be the pancreatic tumor microenvironment (TME), or pancreatic stroma [11]. The TME is composed of both cancer cells and other types of cells that make up the stroma (Figure 1), including stellate pancreatic cells (PSC), cancer-associated fibroblasts, immune and endothelial cells [12]. Additionally, TME encompasses the extracellular matrix proteins (ECM), along with other proteins produced by its cells. The interaction between cancer and stromal cells has been considered an important factor in cancer progression. Abundant stroma is considered to be a distinctive marker of pancreatic cancer, which contributes to the production of growth factors, extracellular matrix protein secretion and fibroblast activation [13]. A study comparing pancreatic cancer cell lines growing in different tumor microenvironments, using orthotopic tumor models, suggested that there are two processes that occur in the TME that can determine the features and conduct of cancer cells, namely, selection and education. In the selection process, some cancer cells become dominant, because of highly malignant characteristics; in the education part, cells attain a malignant phenotype through interaction with TME [14].

One of the main contributors to stromal variation is embodied by PSC. They are thought to participate in stromal activation and PC development. They exist in the normal pancreatic tissue as vitamin A and fat droplets carriers, but in PDAC, once activated, have the ability to induce aberrant secretion of matrix proteins, such as fibronectin, proteoglycans, laminin and glycoproteins in the ECM [15]. Their myofibroblast-like features lead to fibrosis and deviant desmoplasia [16]. The extreme production of ECM thus leads to a desmoplastic environment, which is responsible for drug resistance [17], aberrant vascular perfusion and decreased nutrient accessibility [18]. Another contributor to tumor progression is represented by cancer-associated fibroblasts (CAFs), which play an important role in tumor-stromal interaction [19]. Interestingly, PSCs are thought to be precursors of CAFs [20], and while CAFs can display both pro and anti-tumorigenic proprieties, they are usually correlated with worse outcomes in PDAC patients [21]. CAFs have been linked to extensive tumor development and metastasis in PC [22]. Due to the continuous cell production which contributes to the impenetrable stroma and the lymphatic collapse which occurs in the center of the tumor, up to 80% of the blood vessels in PC are non-functional, bordered by a dense layer of pericityes, thus further impeding drug accumulation [23].

The main pathways involved in development and maintaining the abundant desmoplasia appear to be the vascular endothelial growth factor (VEGF) pathway, which then activates the Ras/Raf/Mek and phosphoinositide 3 kinase (PI3K)/Akt/mTOR pathways; their initiation promotes tumor proliferation, survival and metastasis [25]. A meta-analysis of clinical trials involving stromal targeting agents in pancreatic cancer metastasis [25] found that most trials (51) were directed to angiogenesis, with half of them (26/51) including bevacizumab (anti-VEGF agent), although, unfortunately, several phase II and III trials showed no benefit for its use. Another important pathway in PC is the Hedgehog (Hh) pathway; it appears that it’s ligand, Sonic Hedgehog (Shh) is highly expressed in over 70% of PC cell lines. Olive et al. used mouse models to prove that administering saridegib, an Shh inhibitor, can lead to augmented gemcitabine delivery [26]. However, saridegib is still only used in clinical trials. These results prove that there are still challenges in developing a TME targeted therapy.

### 2.2. Nanotechnology and Targeted Therapy

**Figure 2:**
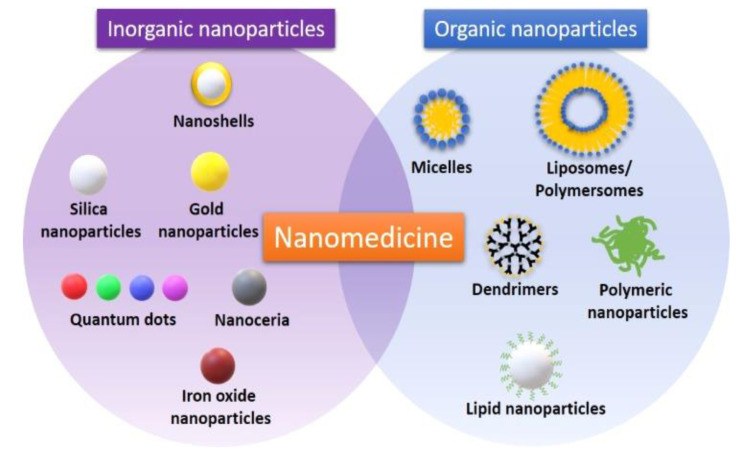
Examples of organic and inorganic nanoparticles used in the field of nanomedicine. Reprinted with permission from [32].

Nanomedicine involves the use of inorganic nanoparticles, such as gold, silica, iron oxide nanoparticles and organic ones, including micelles, polymeric or lipid nanostructures (Figure 2). Nanoparticles have many qualities like a small size (they represent the billionth portion of a meter), low toxicity, they have the ability to be used for targeted therapy and their surface can be adapted for better cell interaction, making them of great value for improving diagnosis and therapy in cancer [27]. As nano-sized transport vehicles, they have overcome many barriers and are of utmost importance in the era of precision medicine. They have been demonstrated to passively mount up in different types of tumors due to the enhanced permeability and retention (EPR) effect [28], or they can actively interact with tumor cells using ligands [29].

In pancreatic cancer, the EPR effect is insufficient, due to dense stroma; therefore, remodeling the TME is necessary to improve drug delivery and nanoparticle distribution. Generally, TME characteristics favor nanoparticle accumulation, but restrict their distribution and extravasation; so far, researchers have tried to overcome these obstacles by influencing tumor vasculature, tumor stress levels or degradation of extracellular matrix [30,31].

Clinical trials involving different types of nanoparticles, such as nanoparticle albumin bound paclitaxel (nab-paclitaxel), gold and micelle nanoparticles, or nanoparticles containing a retroviral gene for targeted therapy in PC have shown promising results so far [33]. Nab-paclitaxel in combination with Gemcitabine has even been approved in 2013 as a first line treatment in metastatic PC, for patients who are not eligible for other, more aggressive therapeutic options; it modestly improves survival by 1.8 months compared to gemcitabine alone [34]. Liposomal irinotecan was also approved in 2016 for patients with metastatic PC, although this treatment also comes with high toxicity [6]. Rexin-G, a gene therapy vector, was used in combination with nanoparticles, in several Phase I/II trials, for treatment of metastatic pancreatic cancer. The grouping showed good results, with improved survival and no organ toxicity [35,36,37]. Micelle nanoparticles encapsulating paclitaxel were used in metastatic PC in a phase I trial, showing an improved anti-tumor activity due to the EPR effect [38]. A nanoparticle composed of liposomes and cisplatin (lipoplatin) in combination with gemcitabine was used for patients with refractory PC, showing a median survival rate of four moths [39]. Generally, these trials have demonstrated that nanoparticles used in combination with chemotherapy is a safe therapeutic option, with low toxicity and great improvement of tumor targeting.

Researchers have also implemented the use of nanoparticles for tumor microenvironment remodeling. Han et al. [40]. established a system based on PEGylated polyethylenimine gold nanoparticles, together with all-trans retinoic acid, which has the ability to lead to PSC quiescence and siRNA targeting heat shock protein 47, which leads to CAFs quiescence, obtaining an increase in the efficacy of gemcitabine treatment by TME modeling. Cun et al. [41]. Developed a combination of size-switchable dendrigraft poly-l-lysine nanoparticles with Gemcitabine (DLG/GEM) and 18β-glycyrrhetinic acid loaded poly(ethyelene glycol)-poly(caprolactone)(PP/GA) for down-regulating CAFs. In addition to remodeling TME, the group also succeeded to enhance the tumor penetration of GEM, with a superior anti-tumor activity, compared to controls. In another attempt to improve drug delivery by surpassing the abundant stroma of PDAC, a team of researchers used collagenase loaded liposomes as a pre-treatment, following then a treatment with paclitaxel loaded micelles; the strategy managed to degrade the ECM and escalate therapeutic effect on a mouse model of PDAC [42].

The use of nanotechnology represents a distinctive prospect for directed distribution of chemotherapy into the tumor cells, improved imaging contrast, these strategies also leading to decreased side effects compared to systemic chemotherapy. These advantages have clear benefit on patient’s quality of life and potentially their survival. Still, there is a clear need for better identification of subjects who might best benefit these therapeutic options, in order to properly develop individualized treatment schemes.

### 2.3. Biomarkers in Pancreatic Cancer

When talking about the early detection of pancreatic cancer, no current study When talking about the early detection of pancreatic cancer, no current study endorses screening asymptomatic patients. Nevertheless, there are certain high-risk categories (patients with hereditary history of pancreatic cancer, hereditary pancreatitis, Peutz-Jeghers syndrome, Lynch syndrome, pancreatic cystic tumors, etc.) for which there are recommendations for early screening [43,44,45]. Furthermore, it is considered that for patients with risk factors (chronic pancreatitis, new-onset diabetes mellitus, obesity, chronic alcohol consumption, smoking) early detection methods should be implemented in order to increase survival [46].

A model diagnostic method for pancreatic cancer should conclusively differentiate malignant from benign tumors, certify accurate tumor staging, and identify early-stage disease and pre-neoplastic conditions. Even though it takes years or decades for PanIN lesions to progress to pancreatic cancer, thus providing a time frame for diagnosis and a prospect for timely management, there are numerous challenges in the early detection of pancreatic cancer, including its asymptomatic nature, lack of specific biochemical tests or imaging variations [47,48].

Primary screening using circulating biomarkers, followed by a confirmatory diagnosis based on imaging and pathological results could be the future strategy for diagnosing PC, although there is still a need for substantial effort in order to overcome limitations present in most studies. First, tumor heterogeneity has been recognized to obscure the chance for an accurate diagnosis. One or two biomarkers can narrowly deliver a comprehensive diagnosis of cancer in the era of precision medicine. Furthermore, selection of suboptimal samples can lead to misunderstanding concerning the diagnostic significance. Most samples in studies were collected from patients with advanced disease rather than from those with early disease. Third, any dynamic changes of biomarkers should be monitored after treatment, during the follow-up protocols, especially in high-risk populations [49,50].

The most pertinent recent techniques for biomarker discovery come from a systems biology approach [51]. Genomic studies, through genome sequencing, polymerase chain reactions (PCR) or fluorescence in situ hybridization (FISH), can lead to the detection of specific genetic biomarkers. Transcriptomics uses microarray profiling and RNA-sequencing for the discovery of expression biomarkers, while proteomics uses mass spectrometry as its main method for proteome characterization. Finally, metabolomics involves mass spectrometry of liquid chromatography for metabolite recognition [52,53].

#### 2.3.1. Carbohydrate Antigens

Presently, there are no validated biomarkers for PDAC detection, carbohydrate antigen (CA) 19-9 remaining the only approved biomarker used for progression and treatment response, but not for detection of pancreatic cancer, due to its low sensitivity and specificity [54]. Furthermore, 5–10% of Caucasians have Lewis-negative blood type, therefore do no produce CA 19-9. Other carbohydrate antigens, including CA 50, CA 72-4, CA 195, CA 242, CEA AND CA-125 have been broadly studied, but none showed superiority to CA 19-9 [55]. A combination of these biomarkers was proposed for better PC detection, but unfortunately, none are standardized or validated [56]. Additionally, an umbrella review of prognostic biomarkers for PDAC highlighted that a combination between CA 19-9 and C-reactive protein to albumin ration (CAR) or CA 19-9 and neutrophil to lymphocyte ratio (NRL) were supported by decidedly suggestive evidence, but the quality of the evidence was generally poor [57].

#### 2.3.2. Growth Factor Receptors

Epidermal growth factor receptor (EGFR) has been one of the most studied receptors for targeted therapy in pancreatic cancer. It belongs to the epidermal growth receptor family, and its activation leads to signaling pathways that promote extensive tumor growth, prompt metastasis and overall high mortality [58]. Erlotinib, an EGFR tyrosine kinase inhibitor, represents the first and only approved EGFR targeted therapy (in combination with gemcitabine), which proved effective in increasing survival in PC [59]. Cetuximab, an anti-EGFR antibody, used in combination with radiotherapy has proved promising results in a phase II study [60].

Insulin-like growth factor-1 (IGF-1) and its receptor are also involved in the development of PC [61], through activating two main signaling pathways: phosphatidylinositol 3-kinase (PI3K)-Akt–mammalian target of rapamycin (mTOR) and RAS/RAF/MAPK thus leading to increased cell survival, proliferation, metastasis and drug resistance. Recent evidence also points to a critical role played by IGF-1 in the development and sustainability of the dense stroma characterizing PC [62], therefore, targeting this growth factor is a valid and promising therapeutic option. Dalotuzumab (MK-0464), a humanized monoclonal antibody directed to IGF-1 receptor, was demonstrated to amplify the gemcitabine effect on PC cells and inhibit the signaling pathways activated through IGF-1. Several phase I or II trials regarding drugs that target IGF-1 signaling were completed, terminated or are on-going [63,64,65,66], and even though there were some encouraging results, the overall outcome is still far from expected.

Transferrin receptor (TfR1) is another membrane protein which appears to be upregulated in over 93% of pancreatic tumors, playing an essential part in the progression of this type of tumor. The pathogenic mechanism behinds these findings is still unclear, but researchers have validated that TfR1 supports mitochondrial respiration and ROS generation in PC, which is indispensable for tumor growth. Given the importance of these studies, TfR1 has become an attractive therapeutic target [67,68].

#### 2.3.3. Mesothelin

Mesothelin (MSLN) is a membrane glycoprotein ordinarily expressed by peritoneum, pericardium or pleural mesothelial cells; studies have shown it can be highly expressed in many types of cancers [69,70]; its involvement in pancreatic cancer has also been summarized in a meta-analysis of 12 studies [71]. It was confirmed that mesothelin is expressed in pancreatic cancer cells, but not normal pancreatic cells, therefore, it could represent a potential biomarker for PC [72]. The meta-analysis also revealed a sensitivity of 0.71 and specificity of 0.88, and suggested that using mesothelin in a combination panel with other biomarkers and a promising new tool for PC detection. Several trials involving complexes such as anti-mesothelin antibody (BAY-94 9343), SS1(dsFv)-PE38 (SS1P is a toxin that targets mesothelin) and MSLN tumor vaccine (CRS-207) have shown potential therapeutic value [73].

#### 2.3.4. Metabolites

A process recognized as the Warburg effect, revealed nearly a century ago, taught us that cancer cells are capable of surviving and proliferating under oxygen and nutrient-deficient conditions [12]. They are also proficient in surviving in these harsh conditions through the process of metabolic reprogramming. This strategy is extremely important for pancreatic cancer, due to their extracellular environment characterized by hypoxia, substantial desmoplasia, and hypovascularization.

Recent technological advances have attracted more attention and interest in cancer-associated metabolic abnormalities and their potential diagnostic and therapeutic applications [74,75]. Accordingly, the uncovering of intermediates in metabolic reprogramming would point out an abnormal biochemical state of a patient and would suggest the existence of a malignancy. Iole et al. showed that serum palmitic acid could differentiate pancreatic cancer patients from healthy controls better than the traditional CA19-9 [76], Kobayashi et al. suggested a combination of four serum metabolites (xylitol, 1,5-anhydro-D-glucitol, histidine and inositol) for detection of chronic pancreatitis and PC, with good results [77]. Leichtle et al. [78] likewise described an association of serum amino acids which were able to discriminate patients with PC and chronic pancreatitis from healthy controls.

Given the important role that metabolic reprogramming plays in pancreatic cancer, including contributing to chemoresistance and radioresistance [79] researchers have tried to develop metabolism targeted therapy. For example, a phase I/II trial involving indoximod, an inhibitor of indoleamine 2,3-dioxygenase (IDO), which is an enzyme expressed in pancreatic cancer, was completed [80]. Another trial involving another IDO inhibitor is recruiting [81]. There are four more trials (recruiting or completed) involving amino acid metabolism targeted therapy in pancreatic cancer, and many others involving all metabolism pathways [79,82,83,84].

#### 2.3.5. Circulating Autoantibodies

Granting little is presently known regarding the origin and pathogenesis behind circulating autoantibodies from the serum of patients with different types of cancer, recently, studies have indicated that they could represent potential biomarkers for the timely detection of cancer. It is already established that autoantibodies against tumor-associated antigens (for example, mutant tumor proteins, overexpressed proteins, or ectopic proteins) are created in numerous types of cancers, counting pancreatic cancer [85,86]. Anti-mucin 1 antibodies (MUC1) have become an important part of autoantibody research in PC. MUC1 is a glycoprotein expressed by the epithelial cells, membrane-bound, that is generally overexpressed in adenocarcinomas, including pancreatic cancer. Gold et al. [87], discovered a monoclonal antibody against MUC1 with 77% sensitivity and 95% specificity when discriminating normal controls from pancreatic cancer. Nevertheless, the diagnostic value of autoantibodies is significantly hindered by tumor heterogeneity.

#### 2.3.6. Matrix Metalloproteinases

Matrix metalloproteinases (MMP) are endopeptidases that have the ability to degrade the extracellular matrix and therefore modulate TME. Their role in pancreatic cancer development has been established: MMP-1 (collagenase), MMP-9 (gelatinase-B), MMP-14 (MT1-MMP) and others are overexpressed in PC and have been proposed as biomarkers for this type of cancer [88]. MMP-14 also appears to be related to gemcitabine resistance [89]. Unfortunately, clinical trials involving MMP inhibitors, such as marimastat did not lead to the expected results; still, due to their essential role in TME behavior, researchers are still trying to develop MMP targeted therapy.

#### 2.3.7. Plectin-1

Plectin-1 (Plec1) is a protein with possible involvement in binding muscle proteins and anchoring microfilaments and microtubules to intermediate filaments. Its potential role as a biomarker for PC has been proposed. A study on mouse models of PC demonstrated that Plec1 was not only expressed in PDAC models, but also in preinvasive pancreatic intraepithelial neoplasia lesions and was also able to differentiate between chronic pancreatitis and pancreatic cancer [90]; another study supported these findings, indicating that Plec1 could also be a potential target for PDAC therapy [91]

### 2.4. Biomarker Targeted Therapy Using Nanotechnology

The field of precision oncology is rapidly expanding, as the use of targeted therapy becomes more advanced. In pancreatic cancer, fast development of different nanoprobes for diagnosis and therapy gives hope for increasing survival of these patients. Conjugation of nanoparticles with various biomarkers in order to increase imaging contrast or tumor accumulation has been tried with promising results (Table 1). It appears that in most cases, the process of conjugating a nanoparticle with a molecule that is overexpressed in PC leads to a more specific treatment method. Even though the biomarkers presented here are not all specific to PC, their use might have potential for clinical implementation.

CA 19-9 targeted therapy using nanotechnology was performed using nanoparticles conjugated with CA 19-9 antibodies and loaded with Paclitaxel (PTX). The complex was associated with ultrasound-mediated microbubble destruction (UMMD), a substance used for increased cellular uptake of the nanocomplex. Results were promising, showing an enhanced therapeutic efficacy of PTX [92].

Based on pancreatic tumor cells ability to overexpress EGFR, researchers have tried using nanotechnology for EGFR targeted therapy, mainly using EGFR’s ligand, EGF. One study proved that by conjugating single wall carbon nanotubes (SWCNT) with EGF, the process will lead to an intense accumulation of the functionalized nanoparticles in a pancreatic adenocarcinoma (Panc-1) cell line [9]. Another study using EGF-conjugated liposomes and curcumin on different pancreatic cancer cell lines, led to an amplification in curcumin effect, namely cytotoxicity and tumor cell death [93].

Magnetic iron oxide nanoparticles (IONPs) were conjugated with recombinant human IGF-1, and along with doxorubicin as the chemotherapeutic, were administered to orthotopic xenograft models of PC. The novelty of the study was also supported by the use of numerous stromal cells in this model. The increased accumulation of the nanosystem was visible by MRI imaging. The study revealed increased apoptosis and inhibition of proliferation of the tumor cells after nanoparticle accumulation. Furthermore, there was no additional toxicity, proving that IGF-1R targeted therapy can represent a promising drug-delivery system [10]. Another team of researchers used SWCNT coupled with IGF-1R antibodies and an imaging agent (CY7) for the photothermal therapy (PTT) of PC. This novel system had a noteworthy curative effect, with minimal side effects, revealing an encouraging new therapeutic approach in the era of precision medicine [94]. Camp et al. used liposomal nanoparticles, conjugated with TfR antibody fragments, loaded with a wild-type p53 gene to improve gemcitabine delivery to PC cells. The p53 gene was used for its antineoplastic and proapoptotic proprieties. The nanocomplex improved the chemotherapy effect also presenting a potential role for gene therapy in this type of cancer [95].

**Figure 3:**
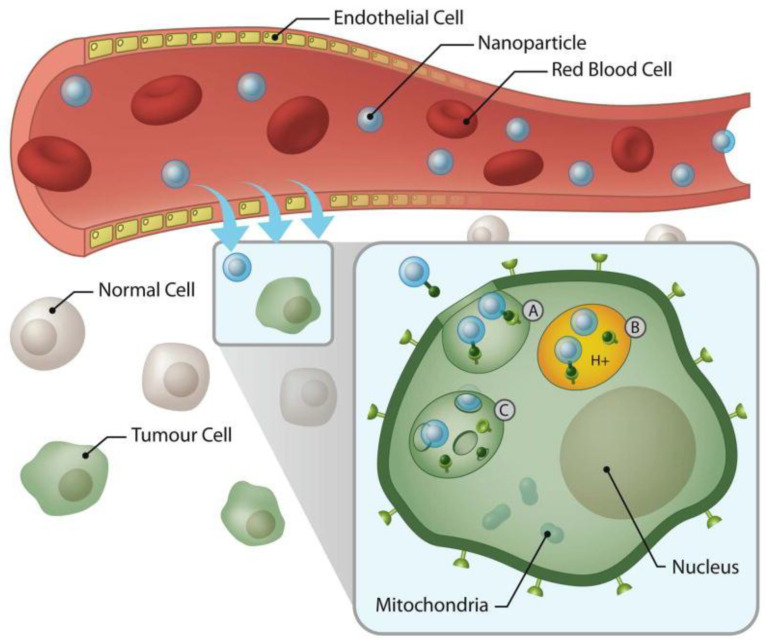
Delivery of magnetic nanoparticles, using active targeting, the process being facilitated the leaky vasculature. A: nanoparticle internalization leading to endosome development; B: High osmotic pressure and swelling of the endosome; C: release of the conjugated nanoparticles. Reprinted with permission from [104].

The increasing demand in new therapeutic options for PC led to great progress in the field of theranostic nanomedicine. Nanoformulations are being now used for simultaneous imaging and therapy. Superparamagnetic iron oxide (SPIO) and ultrasmall superparamagnetic iron oxide (USPIO) are extensively used especially as MRI contrast agents, due to their advantageous characteristics such as contrast potency and low toxicity [103]. Based on these findings, Deng et al. used liposomes loaded with USPIOs, doxorubicin and an anti-MSLN antibody on both Panc-1 cell lines and mouse models of PC. Assembly of the nanoformulation was done by treating the anti MSLN antibody with a reagent and incubating it with the PEGylated liposomes, loaded with the chemotherapy agent and USPIOs (Figure 3). The nanosystem improved imaging of the tumor cells and increased the therapeutic efficacy of DOX, thus, offering a dual benefit for the use of these types of formulations [96].

A team of researchers have tried to manipulate glucose metabolism using nanoparticles in different PC cell lines, afterwards submitting them to photodynamic therapy [105]. Their results were promising, suggesting that a better understanding of the metabolic reprogramming in PC will definitely lead to developing new and improved therapeutic strategies.

Regarding MUC-1 as a biomarker and the use of nanotechnology, superparamagnetic iron oxide nanoparticles were conjugated with underglycosylated mucin-1 tumor-specific antigen (uMUC-1), in order to increase the quality of tumor imaging in treatment follow-up, on a orthotopic model of human pancreatic cancer line. Using MRI and near infrared optical imaging, Medarova et al. [97] demonstrated that the nanocomplex could provide a high-resolution, nonionizing and fast imaging method for detailed assessment of tumor response to PC treatment. A different team of researchers suggested a possible use for magnetic nanoparticles loaded with curcumin on human pancreatic cancer cell lines, by targeting mucin-1. Muc-1 activity in this scenario was reported to have dropped up to 80% after treatment using the nanoformulation described [98]. Zou et al. used SPIO nanoparticles conjugated with MUC-1 in tumor bearing mice. The conjugated nanooparticles managed to increase contrast in MRI imaging both in vivo and in vitro [99].

**Figure 4:**
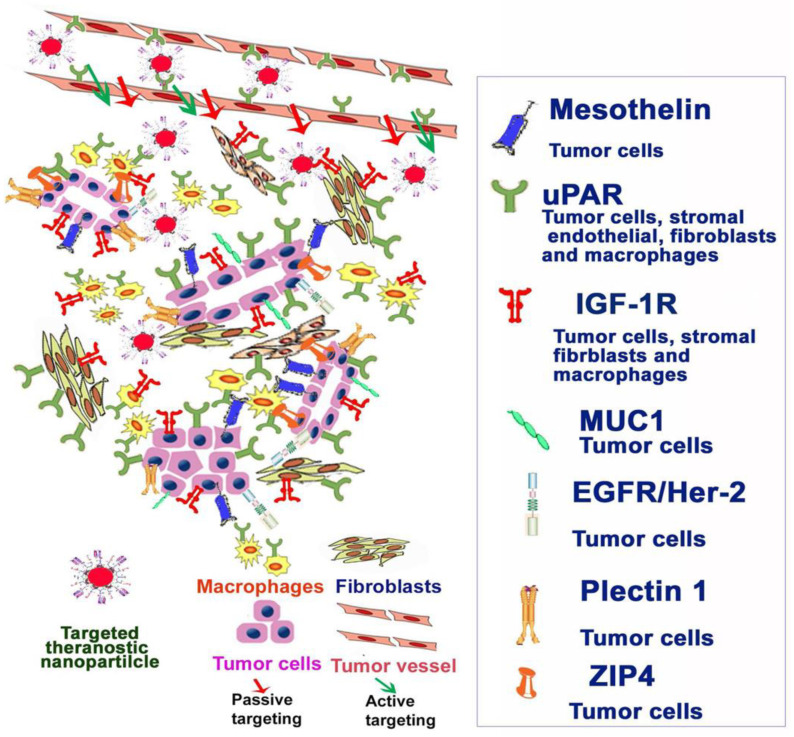
Biomarkers used for their potential role in nanomediated therapy. Abbreviations: uPAR—Urokinase Plasminogen Activator, ZIP4—zinc transporter 4. Reprinted with permission from [105].

Another group of investigators formulated a nanoparticle responsive to MMP-9, that had the ability to trigger gemcitabine release from the nanocomplex in tumor bearing mice in the extracellular matrix of the TME. To ensure proper visualization of the nanoparticles administered, they were infused with carboxyfluorescein and confocal fluorescence microscopy was performed (Figure 4). The authors proved that overexpression of MMP-9 in the TME has the ability to modulate drug release [100].

Wang et al. demonstrated that administration of SPIO nanoparticles along with bovine serum albumin, targeting plectin-1 expressing pancreatic tumor cells improved MRI contrast, revealing a potential role for Plec1 as target for PC imaging [101].Based on these findings, biomarker targeted imaging was also attempted by Chen et al. [102]: they used plectin-1 antibody conjugated SPION nanoparticles and Cy7 as a contrast agent (Plectin-SPION-Cy7) on PC cell lines (MIA PaCa2, Panc-1, XPA-1 and BxPC3) that expressed plectin-1, with MIA PaCa2 and Panc-1 having the highest expression; the probes were then visualized through MRI and confocal microscopy. The study revealed high accumulation of conjugated nanoparticles at the tumor site and improved imaging contrast after administration of the nanocomplex.

As different biomarkers, such as EGFR, IGF-1R, plectin and others like urokinase plasminogen activator or zinc transporter-4 are overexpressed on the tumor cells or on different TME cells, they play a critical role in targeted therapy (Figure 4). The use of nanoparticles in pancreatic cancer is definitively beneficial, and, as studies mentioned above demonstrate, further combining them with potential biomarkers improved therapeutic efficacy and imaging. An extended review on nanomedicine implementation for pancreatic cancer highlights years of research that led to promising results in this field [30]. Our addition is represented by taking one step further and adding specific or non-specific biomarkers onto the nanoparticles used. As PC is highly resistant to chemotherapy, there is a clear need for methods that can overcome this impediment. Researchers proved that by using different nanocomplexes conjugated with biomarkers [9,92,93] the transfer or accumulation of therapy drugs such as curcumin, gemcitabine or paclitaxel in PC cancer cells is augmented; this effect is also useful in reducing systemic chemotherapy toxicity, which often contributes to the low quality of life of the patients. This effect is detrimental for the desired purpose of all oncologic treatments, to improve therapy results with less toxicity. Seeing as a nanocomplex, namely, Abraxane [5], has already been approved for PC treatment, there is hope for other conjugated nanoparticles to be accepted for use.

The use of nanotechnology is also beneficial in manipulating the tumor microenvironment, as highlighted by several reviews [8,22,23], seeing as TME is an important obstacle in drug delivery. Nanoparticle targeted therapy using MMP as a biomarker was successfully used for gemcitabine release in the extracellular matrix of PC [100], strengthening the usefulness of combining therapy methods for augmented results.

Another obstacle in PC therapy is represented by imaging follow up and the use of imaging methods for visualization of the nanoparticles used in order to properly characterize therapy response. The most common nanoparticles used for improving imaging contrast are SPIO and USPIO, with promising results due to their characteristics [96,99,101,102]. Research combined imaging with therapy methods [96,103], further demonstrating the multitude of benefits in using nanotechnology in PC treatment. The use of photodynamic therapy also holds promise for better outcomes in PC [104], but obstacles regarding its side effects and potential toxicity are still an impediment for clinical use [71]. Other potential benefits for improving imaging methods in PC is rapid detection as already mentioned, as it takes years for PanIN lesions to develop into PC, and unfortunately there also no approved biomarkers for early detection. Merging biomarkers with nanoparticles for early detection through imaging methods could represent a novel approach.

The main predicament of all these studies is the lack of clinical implementation due to limitations. There are still unknown factors that need to be taken into account, such as nanoparticle accumulation and elimination, systemic effects, so studies should also focus on understanding the pharmacokinetics of the nanoconjugates used, in order to safely use them.

### 2.5. Prognostic Value of Biomarkers in Pancreatic Cancer

Most patients diagnosed with this type of cancer are not suitable for curative (surgical) treatment and for the ones that are, there are still questions whether there is real and equal benefit for all. [106]. The lack of prognostic tools in PC makes it all the more difficult for clinicians to adhere to the concept of personalized medicine. Still, aside from their crucial role in therapy and targeted imaging, some biomarkers have been demonstrated to play an important part in the prognosis of PC, following surgical treatment. CA 19-9 has limited prognostic value, with its postoperative value seeming more valuable than its preoperative one [107,108]. An immunohistochemical analysis of pancreatic tissues from 137 patients following pancreatic resection revealed that two biomarkers, namely MUC-1 and MSLN had highly prognostic value, predicting survival better than the standard pathologic features used in clinical practice (resections margins, grade, tumor size, lymph node invasion) [109]. Regarding growth factors and their receptors, EGFR status was reported to be associated with the development of metastasis in PC; its high expression was connected to liver metastasis in particular. The study suggested its potential use as a prognostic for metastatic disease [110]. Another study on 122 patients with resected PC found that IGF1R and IGF binding protein-3 (IGFBP3) and their expression is correlated with histological tumor differentiation; immunohistochemical analysis proved that IGF 1 is expressed in advanced stages of PC, while IGFBP3 is downregulated in these stages; these findings suggest a potential use for IGF and its receptor as a prognostic marker for patients undergoing curative treatment [111]. Lin et al. demonstrated the use of transferrin as a prognostic marker for survival in patients with negative CA 19-9 PDAC. A proteomic technique was used in this study to show that Trf was linked with survival and tumor differentiation after curative surgery [112]. All studies showed promising results for better predicting outcomes after therapy in PC patients, but there are impediments to clinical application, as larger cohorts are needed to validate their value.

## 3. Conclusions

Pancreatic cancer remains a disease with poor prognosis, in spite of advances in research. The pancreatic tumor microenvironment plays an essential role in therapy response, cell proliferation, neoplastic development and metastasis and targeted therapy needs to overcome this hurdle as well in order to properly destroy the tumor cells. Nanotechnology, through its numerous advantages, offers faith for developing new and enhanced therapeutic schemes, by permitting nanoparticles to better direct and release chemotherapy medication directly into the tumor site. Biomarkers, besides their important role in the diagnosis and prognosis of any disease, could also play an important part in directed therapy. Most nanoparticle targeted therapy using biomarkers specific or non-specific for PC has shown that by using specific ligands, tumors are better visualized and treated. Granting there is still a long way until clinical implementation, the research done so far has contributed vastly to the advancement in the field of precision medicine.

## Figures and Tables

**Table 1:** Nanoparticles for biomarker targeted therapy and imaging in pancreatic cancer.

Nanocomplex	Nanoparticle	Biomarker Targeted	Effect
PTX-NP-anti CA 19-9 [92]	Three block copolymer organic nanoparticles	CA 19-9	Amplified PC cells uptake of the nanocomplex and drug delivery of Paclitaxel
SWCNT-EGF [9]	SWCNT	EGFR	Increased accumulation in PC cells
EGF-curcumin liposomes [93]	liposome	EGFR	Increased cytotoxic effect of curcumin
IONPs-IGF-1-DOX [10]	IONPs	IGF-1	Improved MRI contrast imaging Augmented apoptosis of tumor cells
SWCNT-IGF-1R antibody-Cy7 [94]	SWCNT	IGF-1	Improved effects of PTT on tumor cells
TfRscFv-Lip-6FAM-ODN [95]	liposome	TfR1	Augmented gemcitabine transfer
Anti-MSLN-PEG-Lipo-USPIO-Dox [96]	liposome	MSLN	Improved MRI contrast imaging Augmented Doxorubicin efficacy
uMUC-1-targeted CLIO-EPPT [97]	SPION	MUC-1	Improved MRI imaging of tumor after treatment
MNP-CUR [98]	Magnetic nanoparticles	MUC-1	MUC-1 activity dropped up to 80%
MUC1-USPION [99]	SPION	MUC-1	Improved MRI imaging
POPE-SS-PEG [100]	liposome	MMP-9	Increase in gemcitabine release at the tumor site
Plectin-SPION-Cy7 [101]	SPION	Plectin-1	Increased MRI contrast imaging and tumor accumulation of the nanocomplex
Plec-1 Ab-SPION-BSA [102]	SPION	Plectin-1	Enhanced targeted imaging
